# Prolyl 3-hydroxylase 1 (P3H1) deficient osteogenesis imperfecta with vascular malformations: a rare disorder with atypical features

**DOI:** 10.1093/jbmrpl/ziag035

**Published:** 2026-03-09

**Authors:** Cherie Chua, Yi Shan Ang, Angeline Lai, Kenneth Wong, Mei Yoke Chan, Mark Koh, Sylvia Kam, Rashida Farhad Vasanwala

**Affiliations:** Department of Paediatrics, Endocrinology Service, KK Women’s and Children’s Hospital, 229899, Singapore; Department of Dermatology, KK Women’s and Children’s Hospital, 229899, Singapore; Department of Genomic Medicine, Genetic Service, KK Women’s and Children’s Hospital, 229899, Singapore; Department of Pediatric Orthopedic Surgery, KK Women’s and Children’s Hospital, 229899, Singapore; Department of Paediatric Subspecialties, Paediatric Haematology/Oncology Service, KK Women’s and Children’s Hospital, 229899, Singapore; Children's Blood and Cancer Centre, KK Women's and Children's Hospital, Singapore; Department of Dermatology, KK Women’s and Children’s Hospital, 229899, Singapore; Department of Genomic Medicine, Genetic Service, KK Women’s and Children’s Hospital, 229899, Singapore; Department of Paediatrics, Endocrinology Service, KK Women’s and Children’s Hospital, 229899, Singapore

**Keywords:** osteogenesis imperfecta, collagen, vascular malformation, P3H1, bisphosphonate

## Abstract

Osteogenesis imperfecta (OI) is a genetic disorder characterized by skeletal fragility with increased susceptibility to fractures, alongside various extra-skeletal manifestations. It has a wide spectrum of clinical presentations and varying degree of severity. Over the years, the classification of OI has expanded, and new pathogenic variants are being identified in genes involved in the regulation of collagen synthesis. We describe a case of a 4-yr-old boy who first presented with right eye swelling, redness, proptosis, and limitation in extraocular movements. Further investigation revealed presence of large left sphenoid vascular malformation (VM). He was treated with repeated embolization and oral propranolol. A year later, he suffered a pathological fracture of the left proximal femur. This was followed by recurrent fractures over a period of 5 yr. Radiographs and MRI showed multiple osteolytic lesions in his skull and extremities, and he had low BMD. Genetic testing identified biallelic variants in the *P3H1* gene. Intravenous zoledronate therapy was commenced and improvement in BMD and remodeling of bones were seen after 4 doses. There was concurrent development of a right sphenoid VM and widespread intraosseous slow-flow VMs seen on serial MRI. He was treated with oral sirolimus for 5 yr and 5 mo duration. There was eventual resolution of sphenoid VMs, while intraosseous VMs remained stable. This is a case of P3H1 deficient OI with atypical clinical features, and the presence of vascular malformations that may or may not be related to underlying collagen defects but may have implications on his treatment and surveillance.

## Introduction

Osteogenesis imperfecta (OI) is a genetic disorder that is primarily characterized by skeletal fragility with increased susceptibility to fractures, alongside various extra-skeletal manifestations. The original classification proposed by David Sillence in 1979 was largely based on clinical severity—ranging from the most severe and lethal Type II OI that results in extensive intrauterine and perinatal fractures, to mild Type I OI that presents with minimal skeletal dysplasia, and few or no fractures. This classification has since been expanded and it is now recognized that OI has a wide spectrum of clinical presentations depending on the underlying genetic variant and molecular mechanisms. Most OI cases are autosomal dominant in inheritance, and result from variants in the *COL1A1* and *COL1A2* genes which encode the proalpha-1 and proalpha-2 chains of type 1 collagen respectively. More recently, new pathogenic variants have been identified in genes involved in the regulation of collagen synthesis, such as *CRTAP* (cartilage-associated protein) and *P3H1*.^1^

P3H1 plays an important role in the post-translational modification of collagen.^2^ It catalyzes hydroxylation at Proline 986 site on alpha-1 chain of Type 1 collagen. Under normal circumstances, different degrees of hydroxylation occur in different tissues^3^—including the bones, tendons, skin, lung, ears, eyes, and kidneys. Reduced or absent P3H1 activity leads to failure of collagen homeostasis and the resultant clinical features of OI.^4^

While biallelic variants in *P3H1* are known to cause severe or lethal forms of OI, there have been more recent reports of patients with compound heterozygous mutations in *P3H1* who display milder phenotypes of OI, suggesting variability in clinical features and severity of disease.^5–7^

In this paper, we describe a case of P3H1-deficient OI with atypical clinical and radiological features, including presence of vascular malformations (VMs). We discuss the molecular mechanisms, clinical course, and treatment outcomes of the case.

## Case presentation

Our patient is a Chinese male born at term via normal vaginal delivery with birth weight of 2730 g. Antenatal course was uneventful and there was no fetal abnormality detected on scans. After birth, he was noted to have bilateral postural congenital talipes equinovarus which improved with physiotherapy. There was no other post-natal concern.

He is the only child to non-consanguineous parents. There was significant history of previous fetal anomaly in an earlier pregnancy for which his mother underwent mid-trimester termination of pregnancy, but no other family history of bone-related or genetic disorders.

**Figure 1:**
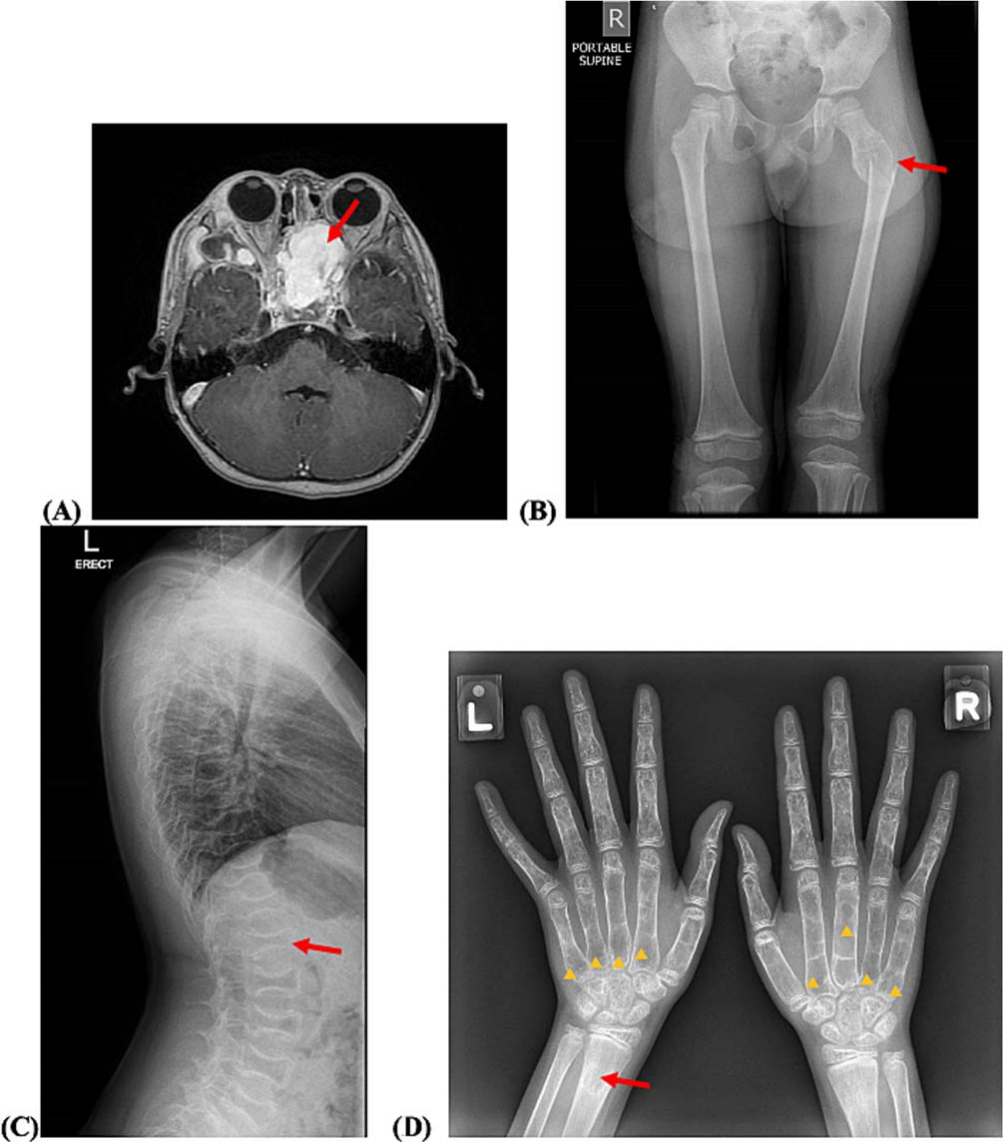
(A) MRI orbits demonstrating left sphenoid vascular malformation (arrow) at initial presentation. (B) Radiograph (antero-posterior view) of bilateral hips demonstrating patient’s first left proximal femur fracture with an underlying expansile lytic lesion (arrow). (C) Radiograph (lateral view) of the spine demonstrating central narrowing of vertebral bodies giving a “fish-like” appearance. (D) Radiograph (antero-posterior view) of bilateral hands demonstrating multiple lucent lesions with cortical irregularity and osteopenia (arrowheads), a lucent lesion in left distal radius with narrow zone of transition (arrow), and a healing fracture over the right distal radius.

He first presented at 4 yr 2 mo old with right eye swelling and redness. Ophthalmologic examination showed right eye proptosis and medial displacement with limitation of movements in lateral, upward and downward gazes. Visual acuity was normal. There was no neurological symptom. Computed tomography of the orbits revealed a large highly vascular heterogenous mass centered in the sphenoid sinus that measured 4.1 × 4.6 × 3.7 cm (main bulk). The mass completely replaced bilateral sphenoid sinuses, extended to the anterior nasal cavity and bilateral ethmoid sinuses, abutted the left maxillary sinus and protruded into the skull base. There was resultant bony remodeling, cortical expansion and thinning of the sphenoid bone, as well as mass effect resulting in compression of the right globe and lateral deviation of the left medial rectus and superior oblique muscles. MRI of the paranasal sinuses yielded similar findings (Figure 1A). Overall, radiological findings favored the presence of an arteriovenous malformation (AVM). The patient underwent three vessels embolization and was started on oral propranolol 2 mg/kg/day. Subsequent scans showed decrease in size of the lesion.

There was no history of bone pain until 5 yr 3 mo old, when he presented with left hip pain after a fall from standing height while walking. X-rays showed a pathological fracture at the subtrochanteric region of the left proximal femur, with an underlying expansile lytic lesion with a narrow zone of transition (Figure 1B). He underwent hip plate fixation with a curettage of the lesion that appeared multiloculated and cystic intra-operatively, with thinning out of the cortices. Histopathological findings were suggestive of a simple bone cyst. There was good healing of the fracture, and the hip implant was removed 18 mo later.

He suffered 2 further fractures subsequently—a right fibula midshaft fracture after being kicked by his peer at 8 yr 7 mo and left thumb metacarpal fracture during sports at 10 yr 6 mo. Both fractures were managed conservatively. During these episodes, evaluation with MRI revealed a well-demarcated homogenous cystic lesion within the medullary cavity of the right fibula that again favored presence of a unicameral bone cyst, and cystic lesions on the right third metacarpal.

At 11-yr-old, he was referred for evaluation of a primary bone disorder.

Physical examination findings were as follows: Height 135.2 cm (SDS −1.37, 8th centile), weight 34.6 kg (SDS −0.36, 37th centile), BMI 18.9 (SDS +0.62, 17th centile), proportionate stature, slight frontal bossing but no facial dysmorphisms, Normal sclerae, teeth appeared normal, no arachnodactyly or brachydactyly, exaggeration of lumbar lordosis, no scoliosis or kyphosis, and no evidence of joint hypermobility. Rest of systemic examination, including his neurology, were unremarkable. He was prepubertal.

**Figure 2:**
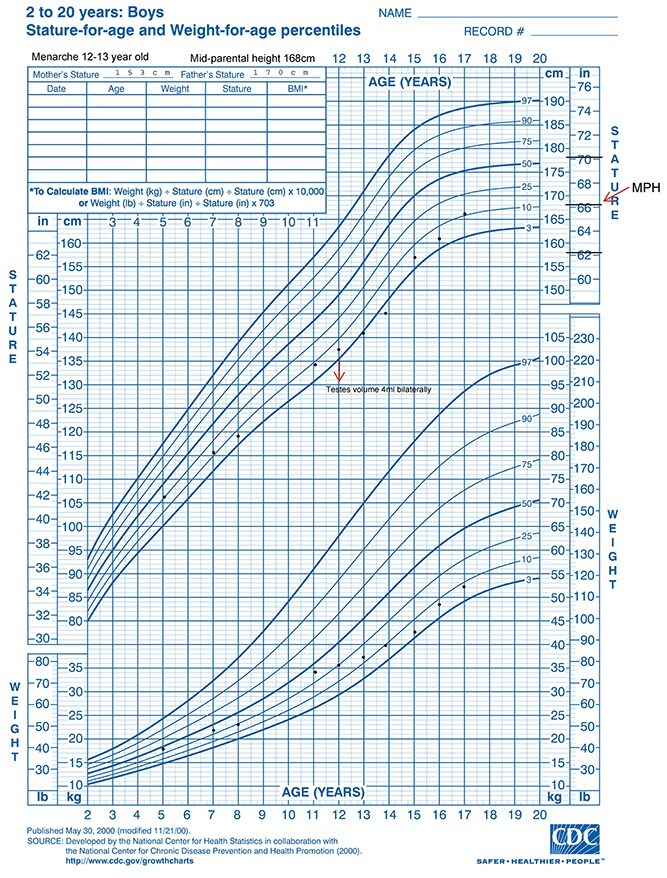
Patient’s growth chart.

Patient’s growth chart can be found in Figure 2.

Putting together this patient’s clinical issues—he had multiple osteolytic lesions complicated by pathological fractures and a large sphenoid VM. It was unclear if these two conditions were related to each other. Further investigations were performed to characterize his clinical phenotype.

### Diagnostic evaluation

Radiographic findings of the lytic lesions were consistent with a benign pathology—well-demarcated margins, narrow zones of transition, lack of aggressive periosteal reactions, non-invasive into surrounding soft tissue. As such, the differential diagnoses for the bone lesions included: simple bone cysts, aneurysmal bone cysts, polyostotic fibrous dysplasia, as well as Maffuci syndrome which is characterized by multiple enchondromas and VM (which was a consideration given the sphenoid vascular lesion).

Bone biochemistry results were normal—adjusted serum calcium 2.53 mmol/L (2.30-2.63), phosphate 1.6 mmol/L (1.3-1.9), alkaline phosphatase 432 U/L (150-490), PTH level 2.1 pmol/L, and 25OHD level 25.3 ng/mL (20-100). Full blood count, renal panel, and liver function test were unremarkable.

Skeletal survey radiographs showed patchy lucencies at the skull vault with lucent lesion in the mandible, central narrowing of vertebral bodies at multiple levels (that would be equivalent to Grade 3 severity by Genant’s semi-quantitative method of classification with >40% reduction in vertebral height) with preserved spine alignment (Figure 1C), multiple lucent lesions in metacarpals and phalanges of both hands, and generalized osteopenia (Figure 1D).

MRI of the right calf and hands characterized the lytic lesions with well-demarcated thinly sclerotic margins, septations and air-fluid levels, hypointensity on T1-weighted images and some hyperintensity on T2-weighted images. There was no cortical destruction, no soft tissue invasion or edema, no ground glass appearance (as in fibrous dysplasia), nor isointensity with muscle tissue on T1-weighted images (as in fibrous dysplasias and enchondromas).

BMD of lumbar spine (L-spine) and total body less head (TBLH) assessed at 13 yr 11 mo old demonstrated low BMD with respective height-adjusted z-scores of −2.11 SD and −4.44 SD.

Histopathology from biopsies of the left proximal femur and left metacarpal bone showed presence of some fibroblasts and fibrous tissue, but there was no replacement of bone with disorganized fibrous tissue typical of fibrous dysplasia, no calcified chondroid matrix typical of enchondromas, nor giant or spindle cells suggestive of aneurysmal bone cysts or giant cell tumors, no histiocytes and no malignant cells.

Genetic testing [Trusight One Panel (Trio)] identified 2 variants in the *P3H1* (Prolyl 3-Hydroxylase 1) gene (also known as *LEPRE1*), both validated by Sanger sequencing. Patient had a paternally inherited variant c.2318C>T (p.Thr773Ile) and a maternally inherited variant c.1517C>G (p.Pro506Arg), according to the American College of Medical Genetics guidelines.^8^ Based on American College of Medical Genetics (ACMG classification), the paternally inherited variant is likely benign with 278 homozygotes reported in gnomAD (BS2_Supp) and a REVEL score of 0.09 (BP4_Strong); while the maternally inherited variant is of uncertain significance with 278 homozygotes reported in gnomAD (BS2_Supp) and REVEL score of 0.78 (PP3_Mod).

High quality sequencing data of the exons of P3H1 were obtained (using Nanopore platform, across all five amplicons with coverage ranging from 4500× to 15 000×) and no other structural or single nucleotide variants of P3H1 were identified. An additional 288 genes associated with OI that are included in Trusight One Panel were reanalyzed, and no other clinically significant variants were detected (including *ANO5, ATP6V062, BMP1, COL1A1, COL1A2, CRTAP, DDX58, FKBP10, GORAB, IFIH1, IFITM5, LRP5, MBTPS2, MESD, PLOD2, POLR3A, PPIB, PYCR1, SERPINF1, SERPINH1, SP7*, and *XYLT2*).

Functional analysis performed on a skin biopsy demonstrated 50% hydroxylation at Proline 986 site on alpha-1 chain of Type 1 collagen, indicating that there is reduced 3-hydroxylation of the Type 1 collagen as this site is typically 100% hydroxylated in normal tissue.

This patient was eventually diagnosed with P3H1-related OI (OI Type VIII).

### Treatment and Progress

#### For osteogenesis imperfecta

Intravenous bisphosphonate therapy was initiated given the low BMD and recurrent fractures. At 14 yr 5 mo old, he received the first dose of zoledronate 0.05 mg/kg given in 2 divided doses, over 2 d. Subsequent zoledronate doses were given as a single infusion of 0.05 mg/kg, in 6-9 mo intervals. He tolerated all infusions well with no adverse effects such as acute phase reactions or hypocalcemia. Prior to his first infusion, patient sustained fractures at his left distal radius and right proximal humerus during physical activities. While on bisphosphonates, he suffered 2 further fractures—right third metacarpal and right proximal humerus (recurrence). These fractures were managed conservatively. He was also maintained on 25OHD at 1000 units daily throughout.

Follow-up DEXA scan performed after 4 doses of zoledronate showed improvement in BMD (Table 1). His lateral spine X-ray demonstrated biconcave appearance of the vertebral bodies but with interval doubling of the vertebral heights, suggesting that there has been remodeling of the vertebrae with bisphosphonate treatment.

**Table 1:** BMD height adjusted *z*-scores.

	Baseline	After 4 doses of zoledronate
**L-spine**	−2.11 SD	+0.68 SD
**TBLH**	−4.44 SD	−2.76SD

Abbreviations: L-spine, lumbar spine; TBLH, total body less head.

#### For sphenoid vascular malformations

Although there was initial reduction in size of the sphenoid VM with improvement of right eye proptosis after vessel embolization and oral propranolol, surveillance MRI performed 4 yr later while on tapering dose of propranolol showed an increase in the size of the sphenoid VM. Propranolol dose was then increased to 3 mg/kg/day. The VM resolved 2 yr later when patient was 10-yr-old.

At 10 yr 10 mo, a new vascular lesion developed in the right sphenoid wing that was not amenable to biopsy or embolization. Despite propranolol therapy, it increased in size and resulted in right eye compressive optic neuropathy. Oral propranolol was stopped and switched to oral sirolimus. There was subsequent resolution of this lesion; however, the patient lost right eye vision by 13 yr of age.

Subsequent follow up scans while patient was on sirolimus showed new cystic lesions on his skull vault, skull base orbits, mandible, and upper cervical vertebrae. Multiple serpiginous structures (appearance of tortuosity) with contrast enhancement and flow voids suggestive of intra-osseous slow-flow VMs with no intra-spinal extension were seen at various vertebrae bodies These remained stable and oral sirolimus treatment was stopped at 16 yr 6 mo old.

## Discussion

Biallelic variants in *P3H1* are known to cause severe or lethal forms of OI-affected individuals typically have severe osteopenia, congenital fractures, extreme short stature, rhizomelia, bulbous metaphyses, normal sclera, and only a few have survived to childhood.^4^^,^^5^ However, there have been more recent reports of patients with compound heterozygous mutations in *P3H1* who display milder phenotypes of OI.^6^^,^^7^ Santana et al. described a case of a 5-yr-old boy with moderate OI from compound heterozygous *P3H1* gene mutations (paternally inherited c.1080+1G>T and maternally inherited c.1646T>G).^9^ He had multiple fractures and lower limb deformities noted at birth and received pamidronate therapy from 7 wk of age. He sustained three other post-neonatal fractures up till the time of writing when he was 5-yr-old. Zhytnik et al. identified P3H1 variants in 17 patients, among a cohort of 146 Vietnamese OI patients. Of these 17 individuals, 14 were homozygous and 3 were compound heterozygotes. The phenotypes of these 17 OI patients varied widely, ranging from moderate (multiple fractures with progressive skeletal deformities) to mild (variable number of fractures with minimal skeletal deformities). In addition, one of the families also reported 2 confirmed OI miscarriages, suggesting severe prenatal OI phenotype.^10^

Our patient presented with his first fracture only at 5 yr 3 mo old. He is of normal and proportionate stature, has Normal sclerae, normal teeth, and no hypermobility of joints. As such, OI was not suspected at the time of his first fracture. An unusual feature that distinguished our patient from other reported P3H1-related moderate OI cases was the radiological appearance of his long bones. Apart from generalized osteopenia, his X-rays showed osteolytic lesions with well-demarcated margins. There were also patchy lucencies over the skull, metacarpals, and phalanges. Hence the other differential diagnoses considered were aneurysmal bone cysts, enchondromas, and fibrous dysplasia—but histopathological findings from the bone biopsy did not support these diagnoses.

Given his clinical phenotype, evidence of reduced P3H1 activity through functional analysis, and no other variants identified through high quality long read sequencing, it is likely that there is underlying disruption in collagen homeostasis from one or a combined effect of the two inherited *P3H1* variants. He had no further fractures since his third dose of bisphosphonate and subsequent investigations showed vertebral body remodeling and improvement in BMD after a total of 4 doses. Maintenance therapy with zoledronate is the recommended treatment for him.

Aortic and intracranial aneurysms have been reported in OI patients. Akin to connective tissue diseases such as Ehler Danlos and Marfan’s syndrome, defective collagen in OI can result in weak vessel wall and a predisposition for aneurysm formation.^11^ These patients are at risk of aortic dissection and intracranial hemorrhage. It is important to distinguish between aneurysms and AVM. These are 2 distinct vascular abnormalities—AVM are abnormal tangles of blood vessels that connect arteries and veins without capillary networks, while aneurysms are abnormal bulges or weak spots that develop in the walls of blood vessels, particularly in arteries. To our knowledge, there has been no reported cases of OI patients with AVM. Although the VMs in our patient may not be related to his Type VIII OI, it remains a consideration whether the reduced P3H1 activity and defective collagen homeostasis affected angiogenesis signaling and led to their development.

Apart from the sphenoid AVM, our patient’s MRI brain and spine also revealed multiple serpiginous structures with contrast enhancement that likely represent intraosseous slow-flow VMs. This finding led us to consider if the appearance of patchy lucencies (on top of the generalized osteopenia) on his bone radiographs were due to abnormal intraosseous vasculature, rather than true osteolysis.

There are some unanswered questions to this case.

First, it is uncertain whether the sphenoid AVM and intraosseous slow-flow VMs seen in our patient are related to the P3H1 deficiency or if these are 2 separate conditions coexisting. A deeper understanding of the mechanisms of P3H1-mediated collagen homeostasis and formation may shed light on the possibility of AVM formations in P3H1 deficient state.

Second, while generalized osteopenia is the main finding in most reported cases of Type VIII OI, our patient’s skeletal radiographs and MRI show discrete cystic lesions. Taking into consideration that he has vascular abnormalities, we considered if these cystic appearance within the bones may be due to abnormal intraosseous vasculature, especially since these lesions persisted even after the improvement in BMD and remodeling of vertebral bodies with bisphosphonate therapy.

Third, our patient did not present with typical features of OI, such as disproportionate or extreme short stature, early-onset fractures, and severe osteopenia. Yet he did have multiple fractures after 5 yr of age, until he received bisphosphonate treatment. This may suggest an acquired contributing factor to bone fragility—such as the development of intraosseous VMs—in addition to the underlying mild-to-moderate OI that resulted in the subsequent recurrent fractures. However, it is challenging to prove the presence of widespread abnormal intraosseous vasculature without more comprehensive tissue sampling.

Lastly, it is uncertain whether presence of intraosseous slow-flow VMs seen in the MRI of his spine can affect interpretation of the BMD, and if the true degree of osteoporosis could be less or more severe than what is measured. This carries an impact on whether response to bisphosphonate treatment can be accurately assessed in subsequent bone mineral densitometry scans and influences decision to continue therapy.

## Conclusion

We describe a case of a young boy with recurrent fractures and P3H1-deficient OI, who has recurrent sphenoid VMs alongside more widespread intraosseous VMs. Improvement in his BMD and remodeling of bones were evident after treatment with zoledronic acid. Sphenoid VMs resolved after a series of combination therapy including embolization, oral propranolol and oral sirolimus. The other intraosseous VMs have remained stable in size even after discontinuation of all pharmacotherapies.

## Data Availability

Original data generated and analyzed during this study are included in this published article.
